# Cysteine import *via* the high-affinity GSH transporter Hgt1 rescues GSH auxotrophy in yeast

**DOI:** 10.1016/j.jbc.2024.108131

**Published:** 2024-12-21

**Authors:** Crystal C. McGee, Tirthankar Bandyopadhyay, Cailin N. McCracken, Evan Talib, Courtney E. Patterson, Caryn E. Outten

**Affiliations:** Department of Chemistry and Biochemistry, University of South Carolina, Columbia, South Carolina, USA

**Keywords:** GSH, cysteine, amino acid transport, redox regulation, yeast genetics, iron–sulfur protein, iron metabolism, metal homeostasis, *Saccharomyces cerevisiae*

## Abstract

Glutathione (GSH) is an abundant thiol-containing tripeptide that functions in redox homeostasis, protein folding, and iron (Fe) metabolism. In *Saccharomyces cerevisiae*, GSH depletion leads to increased sensitivity to oxidants and other toxic compounds, disruption of iron-sulfur (Fe-S) cluster biogenesis, and eventually cell death. GSH pools are supplied by intracellular biosynthesis and GSH import from the extracellular environment. Consequently, in GSH-depleted growth media, deletion of the gene encoding the first enzyme in the GSH biosynthetic pathway (*GSH1*) is lethal in yeast. At the other extreme, GSH overaccumulation *via* overexpression of the high-affinity GSH transporter Hgt1 is also toxic to cells, leading to reductive stress. Here, we engineered a yeast strain that combines *gsh1* deletion with *HGT1* overexpression to study the cellular effects of oscillating between GSH-deplete and -replete conditions. Surprisingly, we find that constitutive expression of *HGT1* in *gsh1*Δ cells rescues the GSH auxotrophy of this strain. We also show that addition of cysteine or cysteine derivatives to the growth media is required for this rescue. GSH limitation in yeast causes intracellular Fe overload because of disruption of an Fe–S cluster–dependent pathway that regulates the activity of the low Fe-sensing transcription factors Aft1 and Aft2. Analysis of Fe regulation and other Fe–S cluster–dependent pathways reveals that *HGT1* overexpression partially alleviates the Fe starvation-like response of *gsh1Δ* cells. Taken together, these results suggest that *HGT1* overexpression facilitates import of cysteine or cysteine derivatives that allow limited Fe–S cluster biogenesis to sustain cell growth in the absence of GSH.

The tripeptide GSH (l-γ-glutamyl-l-cysteinylglycine) is the most abundant low molecular weight thiol in eukaryotic and some prokayotic cells that is present at millimolar concentrations. Its ability to serve as a reductant in biological reactions offers protection against toxic compounds, such as reactive oxygen species (ROS), xenobiotics, and heavy metals (1). In addition, GSH plays a critical role in oxidative protein folding, redox signaling, and iron (Fe) metabolism (2, 3, 4). The importance of GSH is highlighted by the fact that depletion of GSH pools is detrimental to the health and viability of eukaryotes. In yeast, low GSH levels result in oxidant sensitivity, mitochondrial dysfunction, disruption of Fe regulation, and limited cell growth (5, 6). At the other extreme, excess GSH triggers the unfolded protein response in the endoplasmic reticulum (ER) and disrupts Fe homeostasis, leading to cell death (2). Hence, the homeostatic maintenance of GSH is critical to cell viability.

In the model yeast *Saccharomyces cerevisiae*, intracellular GSH levels are maintained *via* two mechanisms: intracellular biosynthesis and import from the extracellular environment (Fig. 1*A*). The biosynthesis of GSH occurs in the cytosol *via* a two-step ATP-dependent enzymatic process. In the first step, γ-glutamylcysteine is formed from glutamate and cysteine in a reaction catalyzed by γ-glutamylcysteine synthetase (Gsh1). In the second step, glycine is added to this dipeptide *via* GSH synthetase (Gsh2). Yeast *gsh1Δ* strains are unable to synthesize GSH and are thus inviable without GSH addition to the growth medium, underscoring the essential function of this tripeptide (7, 8). Exogenous GSH and GSSG are imported from the growth medium *via* the high-affinity GSH transporter Hgt1 (9, 10). A *gsh1Δ hgt1Δ* double mutant is inviable in the presence or the absence of GSH in the growth medium, indicating that Hgt1 is the primary GSH–GSSG import pathway (9). As such, overexpression of *HGT1* in media supplemented with GSH leads to overaccumulation of both GSH and GSSG (2, 9).

**Figure 1:**
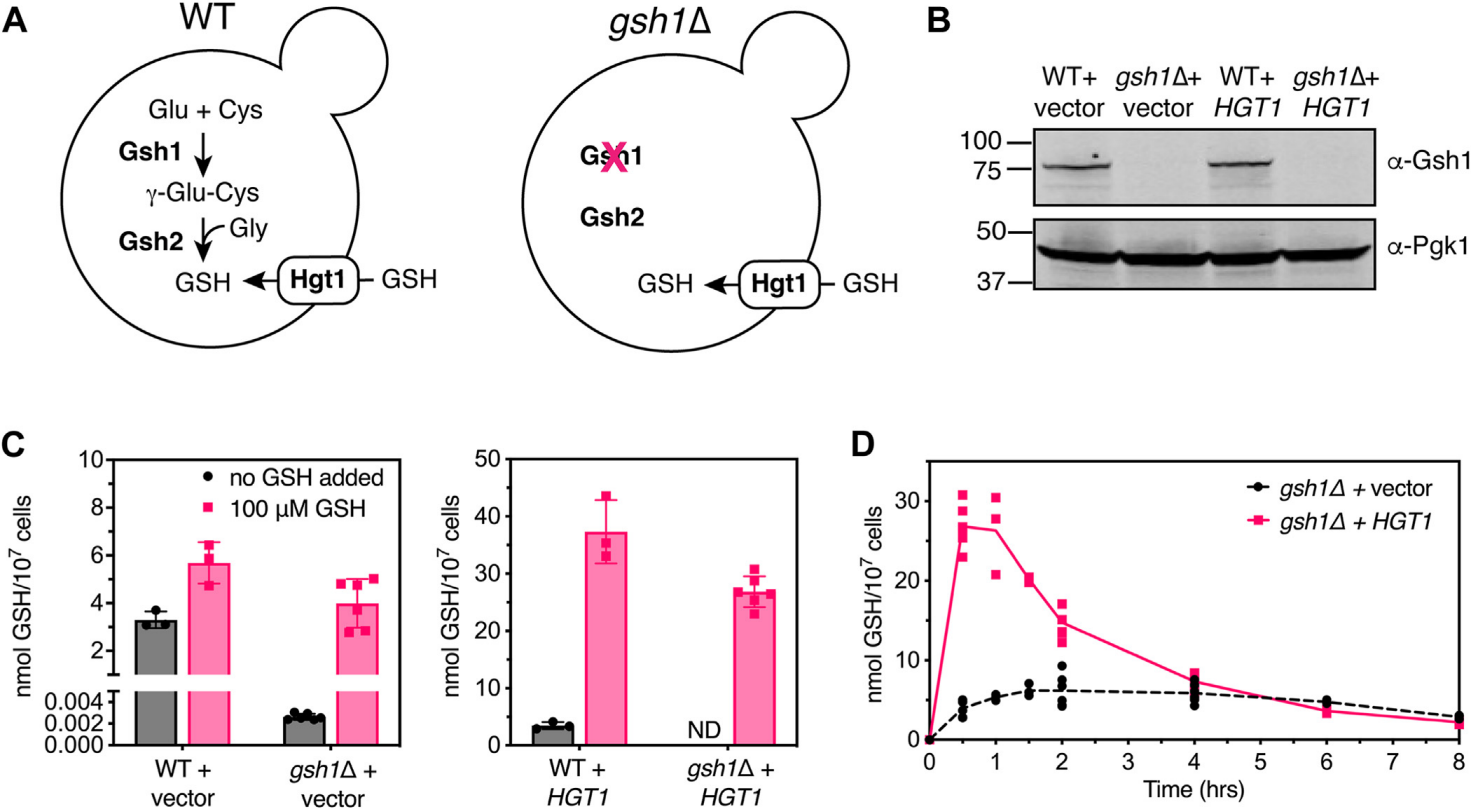
***HGT1* overexpression in WT and *gsh1Δ* strains leads to rapid intracellular GSH accumulation.***A*, GSH biosynthesis and import pathways in WT and *gsh1Δ* cells. *B*, Western blot analysis of whole-cell extracts (75 μg protein loaded) from WT (BY4741) and isogenic *gsh1Δ* strains immunoblotted against Gsh1 and Pgk1 (loading control), confirming deletion of *gsh1*. Positions of molecular mass markers are given in kilodaltons on the *left*. *C*, whole-cell GSH levels in WT and *gsh1Δ* strains transformed with p416TEF vector control (*left*) or p416TEF-*HGT1* (*right*) grown to midlog phase in SC(-Ura) + 0.1 μM GSH. 100 μM GSH (or no GSH) was then added to the media, and cells were harvested 30 min later. The lower *y*-axis is expanded in the *left graph* to show the low but measurable levels of GSH in *gsh1Δ* + vector cells. *D*, time course of intracellular GSH accumulation in *gsh1Δ* strains from (*C*) following addition of 100 μM GSH to SC selection media. Data shown are the means ± SD for three to six biological replicates. ND, not detectable; SC, synthetic complete.

To understand how *S. cerevisiae* viability is impacted by a switch from GSH deplete to replete conditions, we overexpressed the Hgt1 transporter in a *gsh1Δ* strain. Surprisingly, we discovered that GSH-depleted cells overexpressing *HGT1* are viable when grown in synthetic complete (SC) media without GSH supplementation. Measurement of GSH in these strains provided no detectable GSH levels without addition of the tripeptide to the growth medium, suggesting that GSH is not absolutely essential under these conditions. GSH:GSSG redox potential and pH measurements in *gsh1Δ* strains grown with no or low GSH added to the growth media were not substantially different in the presence or the absence of *HGT1* overexpression. However, regulation of Fe metabolism, which is dependent on iron–sulfur (Fe-S) cluster biogenesis, was partially rescued in *gsh1Δ* + *HGT1* cells grown in SC media without GSH. Systematic analysis of SC media components that enabled rescue of *gsh1Δ* + *HGT1* strains identified the amino acid cysteine as a critical factor. Taken together, these results suggest that cysteine imported by *HGT1*, or another cysteine-derived metabolite, may partially substitute for GSH in Fe metabolism to support the viability of *gsh1Δ* strains.

## Results

### *HGT1* overexpression leads to rapid intracellular GSH accumulation in both WT and *gsh1Δ* strains

Our initial goal for this study was to analyze GSH metabolism and trafficking pathways in *S. cerevisiae via* genetic manipulation of yeast GSH biosynthesis and transport. To that end, we created a *gsh1Δ* strain that constitutively uptakes GSH from the extracellular environment *via HGT1* overexpression. Addition of GSH to the growth medium thus allows control of intracellular GSH levels. Deletion of *gsh1Δ* was confirmed by Western blot and whole-cell GSH measurements (Fig. 1, *B* and *C*). Following overnight growth in media with minimal GSH added to sustain growth of *gsh1Δ* strains (0.1 μM GSH), *gsh1Δ* cells exhibit ∼1300-fold lower GSH levels than WT cells (3.3 ± 0.4 nmol GSH/10^7^ cells for WT *versus* 2.6 ± 0.3 pmol GSH/10^7^ cells for *gsh1Δ*), similar to previous reports (2, 11). Addition of 100 μM GSH to the media resulted in a slight increase in intracellular GSH in WT cells (∼1.7-fold) and a substantial increase (∼3500-fold) in *gsh1Δ* cells to levels similar to WT (Fig. 1*C*, *left*). As expected, transformation of these strains with the *HGT1* overexpression plasmid caused a dramatic change in intracellular GSH levels with GSH addition to the growth medium (Fig. 1*C*, *right*). WT + *HGT1* cells exhibited an 11-fold increase in intracellular GSH 30 min after adding 100 μm GSH to the media. Kumar *et al.* (2) previously reported a similar rapid increase in intracellular GSH in WT cells overexpressing *HGT1* after GSH addition, with a gradual decrease to normal levels after 8 h. Interestingly, intracellular GSH was undetectable (<0.5 pmol/10^7^ cells) in *gsh1Δ* + *HGT1* cells after overnight growth in SC with 0.1 μM GSH, unlike *gsh1Δ* + vector cells, which had very low but still detectable levels. However, addition of 100 μM GSH to the media led to a rapid increase in intracellular GSH in *gsh1Δ* + *HGT1* cells, reaching levels similar to WT + *HGT1*. These high GSH levels normalized after 4 to 5 h of growth (Fig. 1*D*), presumably because of degradation of excess GSH (2). Overall, these results confirm the functionality of *HGT1* overexpression in both WT and *gsh1Δ* strains.

### *HGT1* overexpression rescues GSH auxotrophy of *gsh1Δ* cells in SC media and causes increased sensitivity to excess GSH

**Figure 2:**
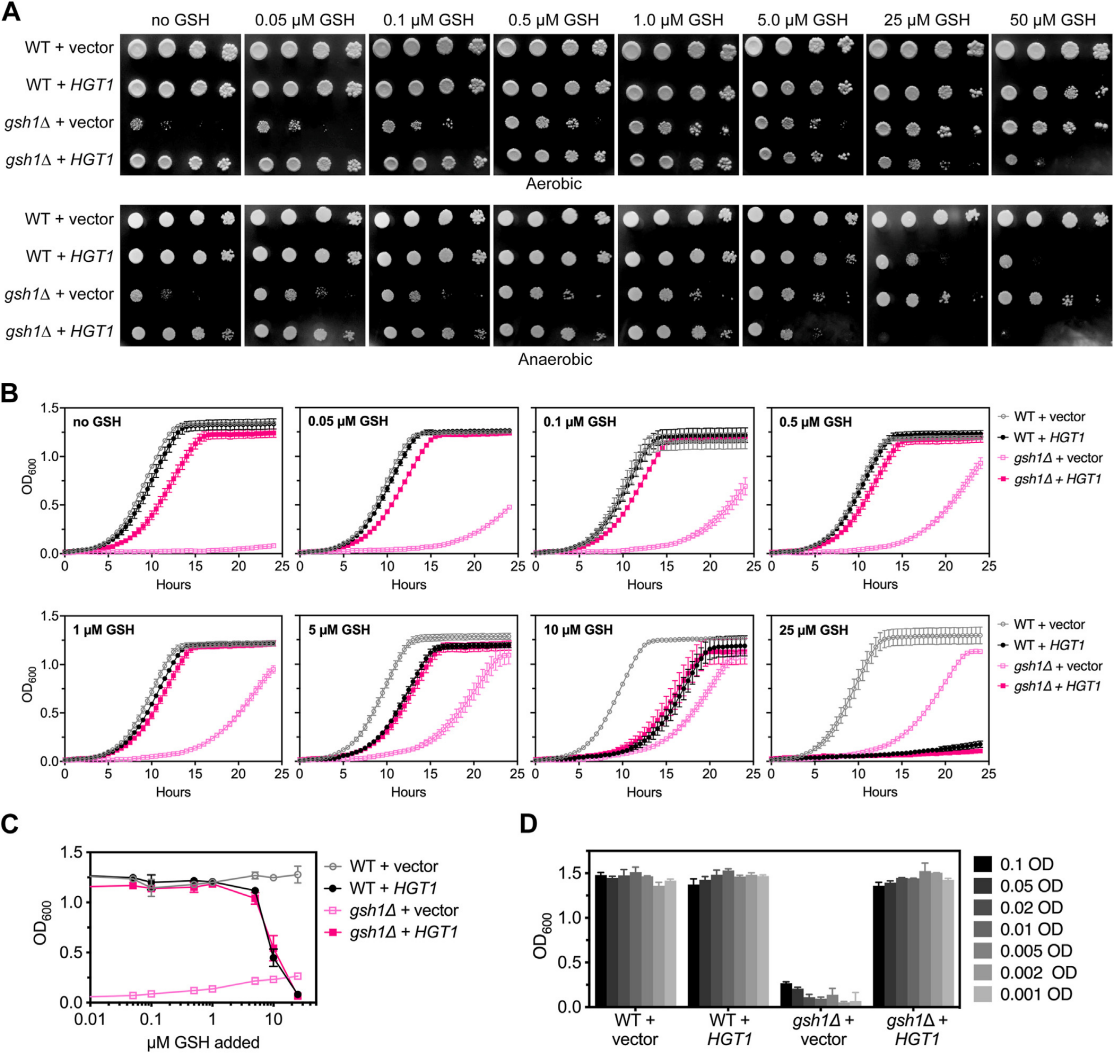
***HGT1* overexpression rescues GSH auxotrophy of *gsh1Δ* cells and causes increased sensitivity to excess GSH.***A*, ten-fold serial dilutions of WT (BY4741) and isogenic *gsh1Δ* strains transformed with empty vector (p416TEF) or *HGT1* overexpression plasmid (p416TEF-HGT1) were plated on SC(-Ura) glucose media with varying GSH concentrations and grown aerobically (*top*) or anaerobically (*bottom*). *B*, the indicated yeast strains were grown in SC(-Ura) glucose media with varying GSH concentrations, and absorbance at 600 nm measurements were taken every 30 min for 24 h. The growth curves are reported as means of three biological replicates ± SD. *C*, absorbance at 600 nm at 15 h growth plotted at a function of GSH concentration from data shown in *B*. *D*, yeast cells were pregrown on SC(-Ura) + 0.05 μM GSH plates for 2 days, inoculated into SC(-Ura) media with no GSH at the indicated starting absorbances, and grown in microplates with shaking for 48 h. Absorbance values represent the mean and SD of two biological replicates. SC, synthetic complete.

We next compared the impact of GSH on the growth and viability of WT and *gsh1Δ* strains with and without *HGT1* overexpression (Fig. 2). Our results confirm a previous report that *HGT1* overexpression is toxic to WT cells when the media GSH concentration exceeds 20 μM (Fig. 2, *A*–*C*) (2). This toxicity is primarily because of inhibition of oxidative protein folding in the ER caused by excess GSH (2). As such, we found that GSH toxicity caused by *HGT1* overexpression in both WT and *gsh1Δ* strains is more pronounced under anaerobic growth conditions in which O_2_ is unavailable to counteract the reductive stress (Fig. 2*A*, *bottom row*). We observed similar phenotypes when adding GSSG to the growth media (Fig. S1), reflecting import of GSSG by *HGT1* and intracellular reduction to GSH by GSH reductase. As expected, *gsh1Δ* + vector cells grew poorly on media with no GSH both aerobically and anaerobically, with growth increasing as [GSH] increased, consistent with previous studies (6). In contrast, *gsh1Δ + HGT1* cells grew surprisingly well in SC media with no GSH added, demonstrating rescue of the GSH auxotrophy. Liquid growth assays revealed a slower growth rate for these cells compared with WT strains; however, *gsh1Δ + HGT1* cells eventually reached similar absorbance values as WT cells in SC media (Fig. 2, *B* and *C*).

Previous reports have shown that *gsh1Δ* cells survive for a limited number of cell divisions when switching to GSH-free media because of GSH accumulation during prior growth in GSH-sufficient media (11, 12, 13). As GSH stores are depleted by cell growth and division, *gsh1Δ* strains eventually reach growth stasis. Thus, it is possible that the enhanced growth observed in *gsh1Δ* + *HGT1* strains in GSH-free media may be due to increased storage of intracellular GSH from the pregrowth media. We thus tested whether inoculating GSH-free media with *gsh1Δ* + *HGT1* cultures at increasingly lower starting absorbances impacted the ability of this strain to grow without GSH (Fig. 2*D*). These data demonstrate that *gsh1Δ* + *HGT1* reach similar final absorbances compared with WT cells regardless of the inoculum size. It is thus unlikely that *gsh1Δ + HGT1* strains store more intracellular GSH than *gsh1Δ* strains.

### *gsh1Δ* + *HGT1* strains do not accumulate and store more GSH than *gsh1Δ* control cells

**Figure 3:**
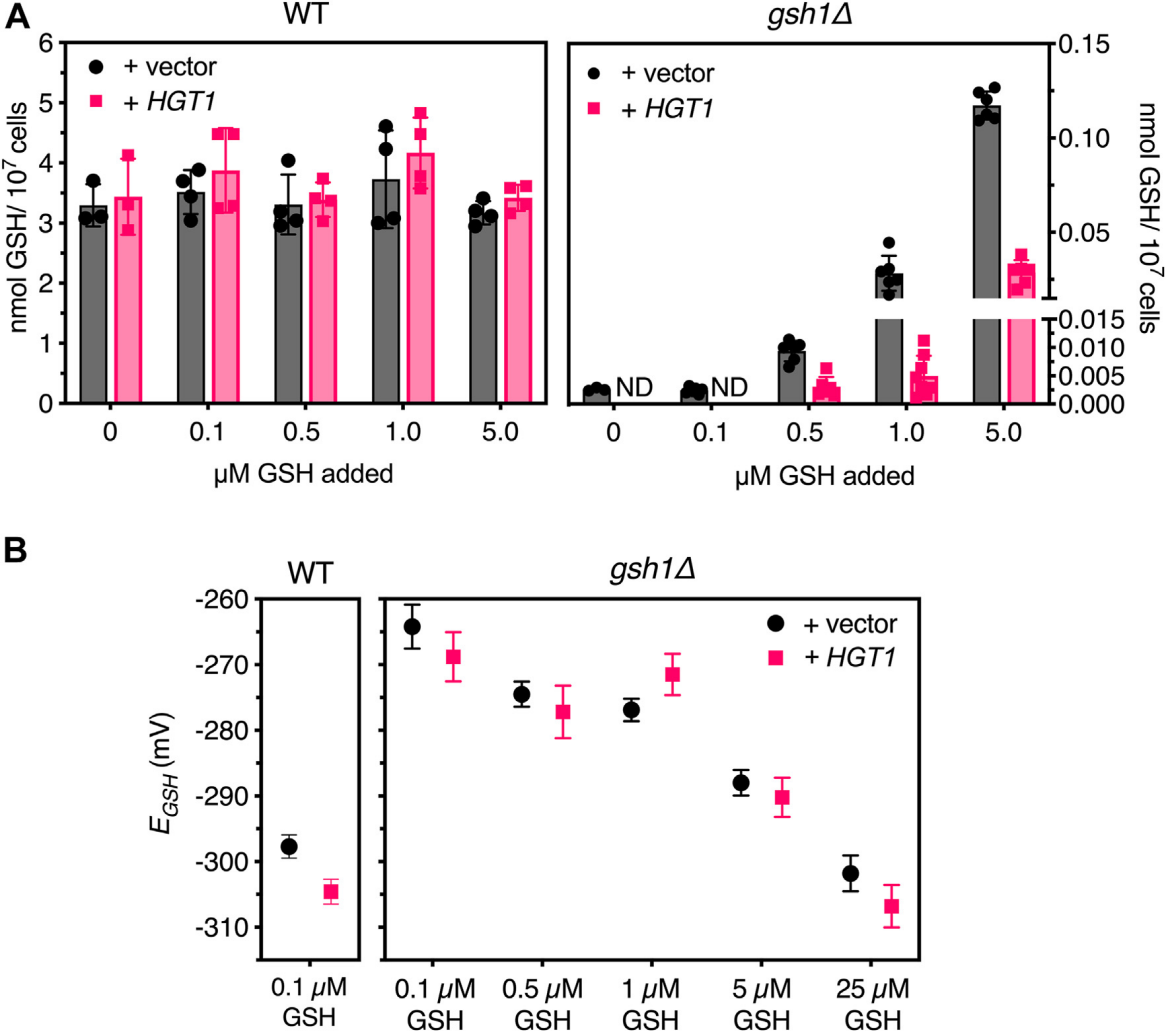
**Overnight growth with increasing GSH in the media leads to less GSH accumulation in *gsh1Δ + HGT1* strains compared with *gsh1Δ* + vector control.** The indicated yeast strains (BY4741 background) from Figure 2 were grown overnight in SC glucose media with the indicated concentrations of GSH to midlog (or growth arrest for *gsh1Δ* + vector). *A*, whole-cell GSH levels in (*left*) WT and (*right*) *gsh1Δ* strains transformed with vector and *HGT1* plasmids were measured. Data shown are means ± SD for three to six biological replicates. ND, not detected because of values lower than the assay detection limit. *B*, GSH:GSSH redox potentials for strains in (*A*) were calculated using the redox state of rxYFP and cytosolic pH measurements for each strain measured with pHluorin (Fig. S2). Data shown are the average and SD of the redox potential calculation. SC, synthetic complete.

To directly compare GSH accumulation and storage in these strains, we grew the yeast overnight in SC media with varying GSH levels. With low GSH concentrations added to the media (5 μM or less), WT cells maintain similar intracellular GSH levels with or without *HGT1* overexpression (Fig. 3*A*, *left*). In contrast, intracellular GSH levels in *gsh1Δ* strains varied with the amount of GSH added and with *HGT1* overexpression. The *gsh1Δ* + vector strain had low but measurable intracellular GSH levels with <0.5 μM GSH in the media, which steadily increased with more GSH added. Interestingly, with *HGT1* overexpression, *gsh1Δ* strains maintained consistently lower GSH levels compared with the *gsh1Δ* + vector control, with no detected GSH with <0.5 μM GSH added to the media (Fig. 3*A*, *right*). These results suggest that with *HGT1* overexpression, *gsh1Δ* cells constitutively import and consume the available GSH at a faster rate than the *gsh1Δ* + vector control. Thus, we conclude that *HGT1* overexpression does not allow enhanced GSH storage in *gsh1Δ* strains but rather has the opposite effect.

We next measured the intracellular GSH:GSSG redox state in these strains using a GFP-based redox sensor to determine how different GSH levels impacted the GSH:GSSG redox potential (*E*_GSH_) in WT *versus gsh1Δ* strains (Figs. 3*B*, S2). Since Hgt1 is a proton-coupled transporter (9, 10, 14) and pH impacts redox potential measurements, we measured the intracellular pH as well using pHluorin, a pH-sensitive GFP-based sensor (15, 16). As expected, the GSH:GSSG redox state of *gsh1Δ* strains was considerably more oxidized (−35 mV difference) compared with WT cells with the lowest levels of GSH added to maintain *gsh1Δ* growth (0.1 μM) (Fig. 3*B*). Increasing GSH levels in the medium progressively lowered the *E*_GSH_ in *gsh1Δ* strains to a similar extent with or without *HGT1* overexpression. Therefore, although there are measurable differences in total GSH levels in *gsh1Δ* strains with or without *HGT1* overexpression (Fig. 3*A*, *right*), this does not translate into substantial differences between the two strains in the GSH:GSSG redox potential (Fig. 3*B*).

### Cysteine import *viaHGT1* is required for growth rescue of *gsh1Δ* cells in the absence of GSH

**Figure 4:**
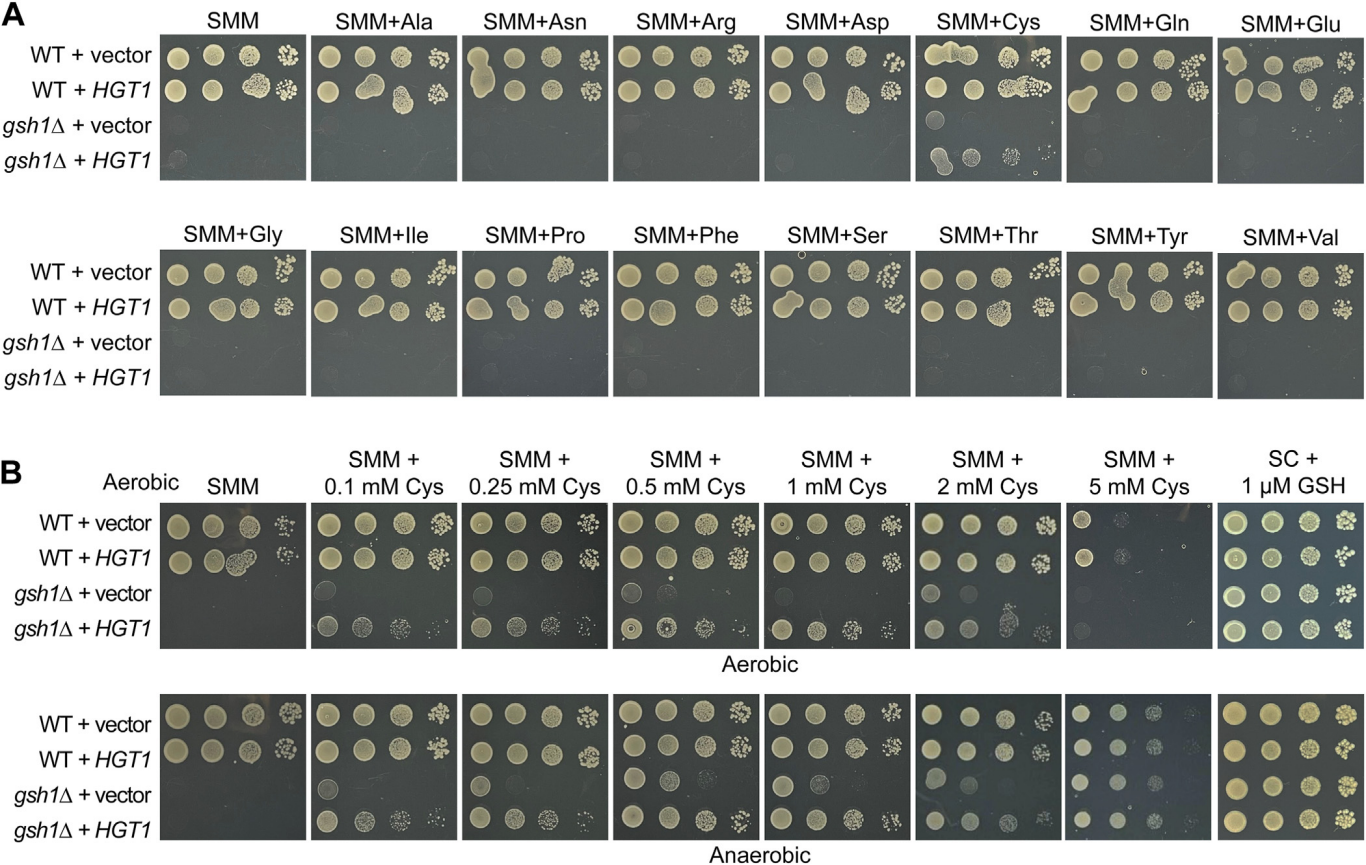
**Cysteine is required for growth rescue of *gsh1Δ + HGT1* strains in the absence of GSH.***A*, ten-fold serial dilutions of WT (BY4741) and isogenic *gsh1Δ* strains transformed with empty vector (p416TEF) or *HGT1* overexpression plasmid (p416TEF-HGT1) were plated on SMM glucose media with the indicated amino acids added (76 mg/l each—equivalent to concentration in SC media) and grown aerobically. *B*, strains in *A* were grown in SMM glucose media with the indicated concentrations of cysteine added to the media. SC + 1 μM GSH plates were included as a positive growth control. SMM, supplemented minimal medium.

We hypothesized that *HGT1* overexpression facilitates increased import of another metabolite in the media that compensates for the lack of GSH, so we spotted these strains in supplemented minimal medium (SMM) that lacks all but six amino acids and bases. The *gsh1Δ* + *HGT1* strain did not grow under these conditions; however, by adding back nonessential amino acids individually, we discovered that growth of the *gsh1Δ + HGT1* strain on minimal media specifically requires cysteine (Fig. 4*A*). Cysteine rescue was effective between 0.1 and 2 mM (for reference, SC media contains 76 mg/l or ∼0.5 mM Cys) but conferred toxicity at concentrations >2 mM under aerobic conditions (Fig. 4*B*), consistent with previous reports (17, 18, 19, 20). This cysteine toxicity was partially alleviated in anaerobiosis, further confirming that cysteine toxicity is primarily driven by increased ROS production (17, 20, 21, 22). Both homocysteine and *N*-acetyl cysteine similarly rescued growth of the *gsh1Δ + HGT1* strain, although homocysteine and *N*-acetyl cysteine were less toxic at higher concentrations compared with cysteine (Fig. S3).

To ensure that GSH was not carried over from the starter plate prior to these growth assays, spot tests were also performed on WT and *gsh1Δ* strains using cells grown for >72 h in liquid media (SC-Ura with no GSH) to deplete any stored GSH. These results confirmed that Cys is sufficient to sustain growth of the *gsh1Δ + HGT1* strain under these more stringent growth conditions (Fig. S4*A*). We tested other common yeast strain backgrounds and observed similar growth rescue for *gsh1Δ + HGT1* cells grown in GSH-free SC and SMM + Cys media (Fig. S4, *B* and *C*). To test whether the functional Hgt1 transporter was necessary for rescue by cysteine, we generated mutants of *HGT1* with reduced transport activity (10, 14). These Hgt1 variants were less effective in rescuing the *gsh1Δ* strain in the presence of cysteine, confirming that *HGT1* transport activity is required (Fig. S5*A*). In addition, we tested whether overexpression of the high-affinity cysteine-specific transporter (*YCT1*) (19) conferred rescue of *gsh1Δ* in the absence of GSH but did not observe the same phenotype as with *HGT1* overexpression (Fig. S5*B*). Taken together, these results suggest that limited cysteine (or cysteine derivative) import by Hgt1 provides an avenue to bypass GSH auxotrophy.

### Defects in Fe regulation are partially rescued by *HGT1* overexpression in a *gsh1Δ* strain

**Figure 5:**
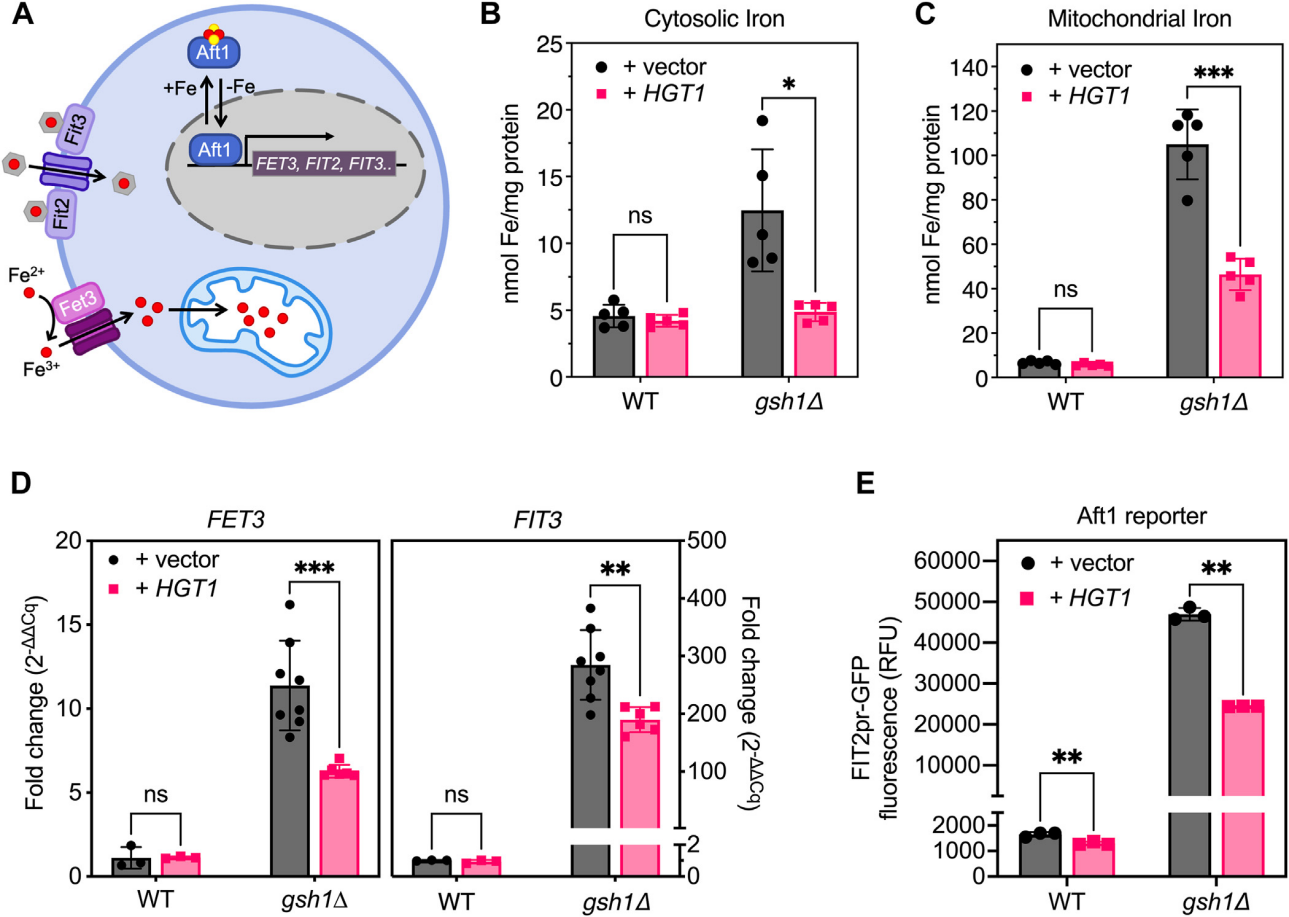
**Dysregulation of iron (Fe) homeostasis in *gsh1Δ* strains is partially rescued by *HGT1* overexpression.***A*, expression of the plasma membrane multicopper ferroxidase Fet3 and siderophore-binding proteins Fit2 and Fit3 is activated by the iron-sulfur (Fe-S) binding transcription factor Aft1 in response to Fe. Fe levels in (*B*) cytosolic and (*C*) mitochondrial extracts were measured by atomic absorption spectroscopy. *D*, quantitative RT–PCR results for *FET3* and *FIT3*. Fold change in *FET3* and *FIT3* mRNA levels is normalized to WT + vector (set as 1.0). *E*, fluorescence measurements of WT and *gsh1Δ* Aft1 reporter strains showing GFP expression driven by the Aft1-regulated *FIT2* promoter. Analyses in *B*–*D* were performed in WT (BY4741) and isogenic *gsh1Δ* strains transformed with p416TEF (vector) or p416TEF-HGT1 (*HGT1*) and grown overnight in GSH-free SC(-Ura) glucose media to midlog (or growth arrest for *gsh1Δ* + vector). Strains in *E* were transformed with p315TEF (vector) or p315TEF-HGT1 (*HGT1*) and grown similarly in low-fluorescence GSH-free SC(-Leu) glucose media. Data shown represent three to eight biological replicates ± SD. SC, synthetic complete.

Since GSH depletion significantly impacts Fe homeostasis in yeast (2, 5, 23, 24), we tested whether *HGT1* overexpression rescued the Fe-related defects observed in *gsh1Δ* strains (Fig. 5*A*). Under Fe deficiency, the primary Fe-responsive transcriptional regulator Aft1 and its paralog Aft2 activate expression of genes required for uptake of ionic Fe (*FET3*, *FTR1*, and *FRE1–3*) and siderophore-bound Fe (*FIT1–3*, *ARN1–4*) at the cell surface to increase intracellular Fe levels (25). When Fe levels are sufficient, Aft1 and Aft2 dissociate from their DNA targets (26), deactivating these genes to prevent Fe overload. GSH depletion disrupts Aft1/2 inhibition in response to Fe (2), leading to intracellular Fe accumulation, especially in the mitochondrion (5). To assess the impact of *HGT1* overexpression on these Fe-related phenotypes, we grew WT and *gsh1Δ* strains ± *HGT1* in SC media without GSH and prepared mitochondrial and cytosolic extracts *via* subcellular fractionation. Analysis of total Fe levels in these samples demonstrates that Fe is increased 2.8-fold and 15-fold in the cytosolic and mitochondrial extracts, respectively, in *gsh1Δ* strains relative to WT strains in the absence of *HGT1* overexpression (Fig. 5, *B* and *C*), consistent with previous reports (5). This Fe overload phenotype is partially rescued in *gsh1Δ* + *HGT1* strains since cytosolic Fe returns to WT levels, whereas mitochondrial Fe is reduced by 63% relative to *gsh1Δ* + vector. To directly assess the impact of *HGT1* on Aft1 transcriptional activity, we measured mRNA levels of Aft1-regulated genes by quantitative RT–PCR (Fig. 5*D*). These results mirror the mitochondrial Fe measurements, showing a significant increase in *FET3* and *FIT3* transcription in *gsh1Δ* + vector cells relative to the WT control, which is partially reduced with *HGT1* overexpression. To confirm this partial rescue phenotype, we also measured Aft1 activity using a reporter strain that expresses GFP from the Aft1-regulated *FIT2* promoter (Fig. 5*E*). These results support the finding that dysregulation of Aft1 activity in response to Fe is partially rescued with *HGT1* overexpression in *gsh1Δ* strains.

### *HGT1* overexpression allows limited Fe–S cluster biogenesis in *gsh1Δ* strains

**Figure 6:**
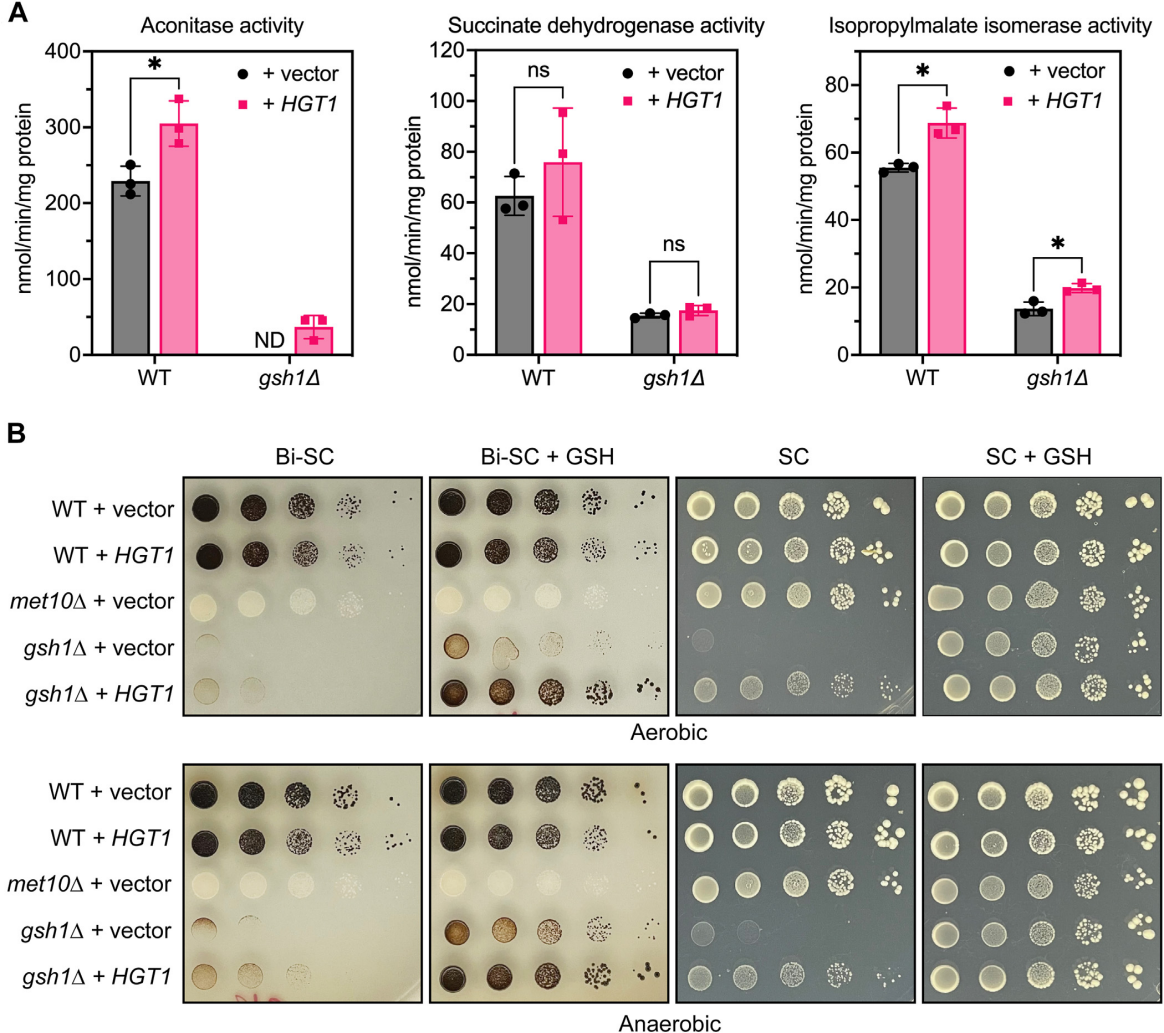
***HGT1* overexpression allows limited iron–sulfur (Fe–S) cluster biogenesis in *gsh1Δ* strains.***A*, enzyme activities of mitochondrial Fe–S cluster enzymes aconitase and succinate dehydrogenase (SDH) and cytosolic Fe–S enzyme isopropylmalate isomerase (Leu1) were assayed in cell lysates prepared from the indicated yeast strains. Data shown are the means of three independent experiments ± SD. For aconitase and SDH assays, WT (BY4741) and isogenic *gsh1Δ* strains were transformed with p416TEF (vector) or p416TEF-HGT1 (*HGT1*) and grown in SC(-Ura) media, whereas for Leu1 assays, these same strains were transformed with p315TEF (vector) or p315TEF-HGT1 (*HGT1*) and grown in SC(-Leu) media. ND, not detected because of values lower than the assay detection limit. *B*, the indicated yeast strains transformed with p416TEF (vector) or p416TEF-HGT1 (*HGT1*) were spotted as serial dilutions on bismuth-sulfite-SC(-Met/-Ura) (Bi-SC) or SC(-Met/-Ura) plates ± 1 μM GSH. The *met10Δ* + vector strain is a control that lacks sulfite reductase activity. *Top row* was grown under aerobic conditions, whereas *bottom row* was grown anaerobically. SC, synthetic complete.

Previous *in vivo* and *in vitro* studies have demonstrated that inhibition of Af1/2 activity in response to Fe is dependent on Fe–S cluster biogenesis (27, 28). This dependence is due to the nature of the regulation mechanism, which involves transfer of a [2Fe–2S] cluster to Aft1/2 by the cytosolic Fe–S cluster chaperones Grx3/4 and Bol2 (formerly Fra2) to facilitate Aft1/2 DNA dissociation (29, 30, 31, 32, 33). To further study the impact of *HGT1* overexpression on Fe–S cluster metabolism, we tested the activity of mitochondrial and cytosolic Fe–S cluster enzymes in *gsh1Δ* strains ± *HGT1* grown in SC media. We note that GSH depletion in *gsh1Δ* strains significantly reduced the activity of both mitochondrial (aconitase and succinate dehydrogenase) and cytosolic (isopropylmalate isomerase) Fe–S cluster enzymes (Fig. 6*A*). However, we observed small but significant increases in aconitase and isopropylmalate isomerase activity with *HGT1* overexpression in both WT and *gsh1Δ* strains. These findings suggest that increased *HGT1* expression may enhance Fe–S cluster biogenesis or minimize Fe–S cluster turnover. We also measured cytosolic Fe–S cluster enzyme activity in these strains *via* a bismuth sulfite plate assay (Fig. 6*B*). Sulfide generated by the cytosolic [4Fe–4S] enzyme sulfite reductase reacts with bismuth to generate the brown precipitate bismuth sulfide. In the absence of GSH on bismuth-sulfite-SC (Bi-SC) plates, the *gsh1Δ* + *HGT1* strain exhibits slightly more brown color than the *gsh1Δ* + vector control strain, suggesting limited but detectable sulfite reductase activity under both aerobic and anaerobic conditions. Addition of 1 μM GSH to the Bi-SC plates restores sulfite reductase activity in both *gsh1Δ* strains, although to a much greater extent in *gsh1Δ* + *HGT1* cells as evidenced by their dark brown color. Taken together, these results demonstrate that GSH depletion significantly impacts the activity of Fe–S cluster enzymes in both the cytosol and mitochondria, whereas *HGT1* overexpression allows limited rescue of some Fe–S cluster enzyme activities in the absence of GSH.

### *HGT1* rescue of GSH auxotrophy is less effective in nonfermentable carbon sources

**Figure 7:**
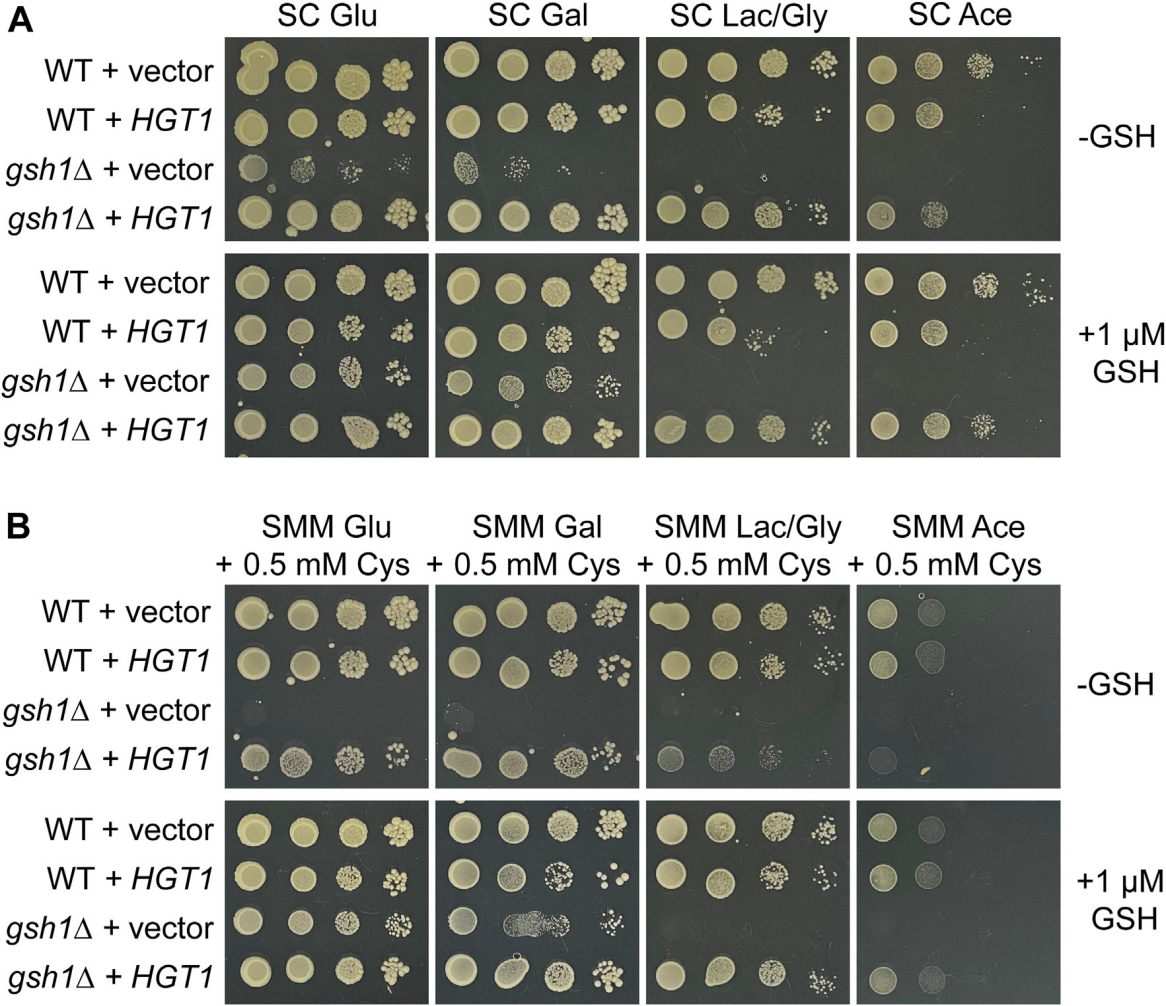
**Growth of *gsh1Δ + HGT1* strains is reduced on nonfermentable carbon sources.** Ten-fold serial dilutions of WT (BY4741) and isogenic *gsh1Δ* strains transformed with empty vector (p416TEF) or *HGT1* overexpression plasmid (p416TEF-HGT1) were plated on SC (*A*) or SMM + 0.5 mM Cys media (*B*) ± 1 μM GSH with the indicated carbon sources and grown aerobically. Ace, acetate; Gal, galactose; Glu, glucose; Lac/Gly, lactate/glycerol; SC, synthetic complete; SMM, supplemented minimal medium.

To further explore the impact of *gsh1* deletion and *HGT1* overexpression on Fe–S cluster–dependent activities, we compared the growth of these strains in carbon sources with different metabolic requirements. As expected, *gsh1Δ* cells showed no growth on nonfermentable media as previously observed (6), even with 1 μM GSH addition. This respiratory incompetency of *gsh1Δ* strains is primarily attributed to Fe-dependent ROS accumulation that impacts the mitochondrial genome stability (6). Compared with the *gsh1Δ* + vector strain, the *gsh1Δ* + *HGT1* strain grew well in SC media with rapid and slow fermentable carbon sources (*i.e.*, glucose and galactose) that rely less on mitochondrial function (Fig. 7*A*). However, growth of the *gsh1Δ* + *HGT1* strain was slightly reduced in GSH-deficient SC media with nonfermentable carbon sources such as lactate/glycerol and acetate. This defect was even more pronounced in the minimal SMM + Cys media (Fig. 7*B*). Energy production by yeast growing on nonfermentable carbon sources requires the tricarboxylic acid cycle and the mitochondrial respiratory chain, which are both critically dependent on Fe–S-binding proteins. Furthermore, mitochondrial Fe levels are still relatively high in the *gsh1Δ* + *HGT1* strain, which may contribute to ROS-induced mitochondrial DNA damage. Therefore, it is likely that cysteine alone at the concentration tested is not able to effectively replace the function of GSH in Fe–S cluster biogenesis required for Fe–S-dependent energy production and Fe regulation under these more demanding growth conditions.

## Discussion

The dependence of eukaryotic cells on GSH for growth and survival has been well characterized in yeast by studying *gsh1Δ* and *hgt1Δ* strains that lack the ability to synthesize and import GSH, respectively. The sensitivity of GSH-depleted strains to oxidants, xenobiotics, and heavy metals is attributed to the function of GSH as a cofactor for antioxidant and detoxification enzymes (1, 6, 8, 11, 34, 35, 36, 37). In addition, GSH impacts oxidative protein folding pathways in the ER and mitochondrial intermembrane space by influencing thiol-disulfide exchange reactions (2, 38, 39, 40, 41, 42). However, these GSH-dependent pathways do not explain the essentiality of GSH for cell survival, which is instead attributed to its role in cytosolic–nuclear Fe–S cluster assembly. Severe GSH depletion (∼100–130-fold lower levels than WT) was found to impair the biosynthesis of cytosolic and nuclear Fe–S cluster proteins that have essential roles in cell survival (2, 5, 43). Several proteins in the Fe–S cluster biogenesis pathway are known to require GSH for their function, including the mitochondrial (Grx5) and cytosolic (Grx3, Grx4) monothiol glutaredoxins and the mitochondrial ABC transporter Atm1/ABCB7. The glutaredoxins use GSH as a ligand to bind and traffic Fe–S clusters in mitochondrial and cytosolic–nuclear compartments (4, 44, 45, 46) but are not absolutely essential for cell survival (30, 32, 43, 47, 48). On the other hand, Atm1 is an essential mitochondrial protein that employs GSH to export a sulfur- and Fe-containing species (known as X–S or [Fe–S]_int_) required for assembly of nuclear–cytosolic Fe–S clusters (5, 49, 50, 51, 52). The exact nature of this Atm1 substrate(s) is unclear, but available crystal structures of yeast Atm1 and a bacterial homolog clearly reveal GSH or GSSG binding in the substrate cavity formed by the dimer interface (49, 51). Therefore, the key GSH-dependent step in cytosolic and nuclear Fe–S cluster biogenesis is proposed to be Atm1 export of X–S/(Fe–S)_int_ (43).

Here, we show that by overexpressing the high-affinity GSH–GSSG transporter Hgt1 in a GSH-deficient *gsh1Δ* strain, we have created conditions in which GSH is no longer essential for cell survival. Without GSH, the *gsh1Δ + HGT1* strain can grow in solid and liquid SC media, unlike the *gsh1Δ* control but exhibits sensitivity to GSH at concentrations >5 μM. Because growth inhibition by GSH upon *HGT1* overexpression is more significant under anaerobic conditions when oxygen is not available to counteract reductive stress, this sensitivity is likely because of unregulated GSH flux providing excess reducing power that disrupts pathways dependent on oxidative protein folding (2, 41). However, at the other extreme, after overnight growth in SC with very low (0.1 μM) or no GSH, the *gsh1Δ + HGT1* strain has no detectable intracellular GSH. Thus, in SC with <0.1 μM GSH added, we suspect that unregulated GSH import by *HGT1* overexpression leads to rapid GSH consumption and depletion from the media as the cells grow and divide.

Since this growth rescue was observed in amino acid–rich SC media but not the low amino acid SMM media, we focused on determining which specific amino acid provides the resource required for growth. Since the function of GSH is dependent on its redox-active thiol group, it is not entirely surprising we identified cysteine as the key factor. We show that cysteine (as well as cysteine derivatives homocysteine and *N*-acetyl cysteine) partially rescues the growth of *gsh1Δ* + *HGT1* cells in SMM. This rescue is less effective with *hgt1* mutants that have impaired transport activity, suggesting that Hgt1 directly imports cysteine. In fact, cysteine has been shown to competitively inhibit GSH transport by Hgt1 (9), suggesting that it can also serve as a substrate for this transporter. Yet, surprisingly, we show that *YCT1* overexpression (encoding a cysteine-specific transporter) was unable to rescue the GSH auxotrophy of *gsh1Δ* cells. This may reflect differences in the kinetics and/or thermodynamics of cysteine uptake by the two transporters, since excess cysteine is toxic to cells at higher concentrations because of its ability to generate ROS (17, 18, 20, 53). The *YCT1* gene is normally upregulated during GSH depletion (54), but constitutive and high-level expression does not appear to be beneficial in this situation. Constitutive *HGT1* overexpression may enable slow and steady low-level cysteine uptake that partially rescues growth without causing toxicity. The reasons that homocysteine and *N*-acetyl cysteine are less toxic than cysteine are unclear, but this phenomenon has been observed previously (17, 18, 20). The distance between the amine and thiol group and the presence of a free amine are likely important factors affecting thiol reactivity. In fact, both homocysteine and *N*-acetyl cysteine are more resistant to oxidation than Cys, especially in the presence of redox-active metals (55, 56, 57). Cys may thus promote ROS generation *via* Fenton chemistry to a greater extent than other aminothiols, leading to increased oxidative stress (58).

To further probe the mechanism of Cys rescue in the absence of GSH, we focused on Fe regulation. Our results confirm that GSH depletion activates the Aft1/2 regulon, reflecting a defect in Fe–S cluster biogenesis that causes an Fe starvation response, which leads to increased intracellular Fe levels. We found that *HGT1* overexpression in Cys-containing media partially rescues this defect, suggesting that the Fe–S cluster–dependent signaling pathway that post-translationally regulates Aft1/2 activity in response to Fe is partially restored. As noted previously, GSH is required at several steps in this signaling pathway, where it serves as a substrate for the mitochondrial exporter Atm1, and as a ligand for Fe–S cluster–binding glutaredoxins in the mitochondria (Grx5) and cytosol–nucleus (Grx3/4) (43, 59). Our findings suggest that GSH is not absolutely essential for the function of these proteins and may possibly be substituted by Cys or a Cys-derived metabolite under certain conditions. This idea is supported by published biochemical activity assays for purified yeast Atm1, demonstrating that Cys strongly stimulates the ATPase activity of this transporter (60), implicating Cys as a potential substrate. Interestingly, the stimulatory effect of Cys on Atm1 ATPase activity is 20-fold higher than GSH (60). However, GSH is typically found at much higher concentrations than Cys in cells (2, 61) and thus is likely the preferred substrate under normal conditions. There is also evidence that Cys can replace GSH as a cluster ligand in monothiol Grxs that deliver [2Fe–2S] clusters to Aft1/2 to inhibit their DNA-binding activity. Purified yeast Grx3 can form [2Fe–2S]-bridged homodimers in the absence of GSH when other low molecular thiols are provided to ligate the Fe–S cluster (*e.g.*, Cys, DTT), and these forms are functional in Fe–S cluster transfer assays (62). In addition, mitochondrial Grx5 has been shown to ligate a [4Fe–4S] cluster in the absence of GSH using an additional semiconserved Cys at the C terminus (63). These previous findings support the idea that the role of GSH in Fe signaling may be bypassed or functionally replaced by other thiol compounds when GSH is absent.

Finally, we investigated the impact of GSH depletion + *HGT1* overexpression on other Fe–S-dependent pathways in yeast *via* enzymatic assays and respiratory growth assays. We found that depletion of GSH dramatically lowered the activity of both mitochondrial and cytosolic Fe–S enzymes and reduced the viability of yeast strains grown on nonfermentable carbon sources. We note that, in contrast to our findings, previous researchers reported that mitochondrial Fe–S enzymes are still functional with significant GSH depletion (reaching 120–130-fold less GSH levels than WT) (2, 5). However, for our experiments, *gsh1Δ* strains were cultured in GSH-free media until growth stasis to fully deplete GSH (typically reaching 1300-fold less GSH than WT) (12). The lack of activity we observed may thus be explained by the extent of GSH depletion used in our experiments compared with theirs and differences in the dynamics of GSH depletion in the cytosol *versus* mitochondria. Previous measurements of the GSH:GSSG redox potential in the cytosol and mitochondria of yeast have demonstrated that cytosolic GSH pools are impacted earlier and more significantly by GSH depletion than mitochondrial GSH pools (6). Therefore, disruption of mitochondrial Fe–S cluster biogenesis likely requires a greater degree of GSH depletion than the cytosolic pathway, as previously suggested (64). In any case, we found that *HGT1* overexpression caused small but detectable increases in the activity of some Fe–S proteins we tested (aconitase, isopropylmalate isomerase, and sulfite reductase). However, it appears that this partial rescue of Fe–S cluster enzyme activity was not sufficient to support robust growth on nonfermentable carbon sources (especially in minimal media), which places a higher demand on the Fe–S cluster biogenesis machinery.

In summary, this study provides valuable insights into the essential nature of GSH in eukaryotic cell metabolism and its connection to Fe–S cluster biogenesis and Fe regulation. By genetically manipulating GSH biosynthesis and import pathways in yeast and testing different growth conditions (varying GSH, oxygen, amino acids, and/or carbon sources), we have revealed a crucial role for cysteine during GSH depletion, presumably by functional replacement of GSH in Fe–S cluster trafficking pathways. These findings have implications for numerous human disorders or disease conditions that are characterized by an imbalance in amino acid, metal, and/or redox metabolism (4, 45, 65, 66, 67). However, further analysis of the flow of metabolites through the trans-sulfuration pathway in this model system as well as exploration of changes in other metabolic pathways in response to GSH depletion combined with *HGT1* overexpression is required to fully uncover the cellular and molecular mechanisms that allow cell survival in the absence of GSH.

## Experimental procedures

### Yeast strains, media, and growth conditions

*S. cerevisiae* strains used in this study are listed in Table S1. Strains were grown in SC selection media (0.67% yeast nitrogen base with ammonium sulfate [US Biological Y2025]), 2% glucose, supplemented with the appropriate drop-out mix (US Biological) or in supplemented minimal media, termed SMM (0.67% yeast nitrogen base with ammonium sulfate, 2% glucose, supplemented with amino acids and bases at the following concentrations: 20 mg/l adenine, 100 mg/l leucine, 30 mg/l lysine, 20 mg/l histidine, 20 mg/l tryptophan, 20 mg/l uracil, and 20 mg/l methionine). Alternative carbon sources used for some plates included 2% galactose, 2% glycerol/2% lactate, and 2% acetate. Strains used for Aft1 reporter assays were grown in low fluorescent SC drop-out media (Sunrise Pharmaceuticals). Other modifications of these media recipes are noted in the text. We note that the brand of agar using in preparing solid SMM plates and the age of the plates sometimes affected the growth phenotype for *gsh1Δ* strains. This phenomenon has been observed previously in other yeast research laboratories and is attributed to sporadic toxicity in specific agar batches (68). Therefore, to maintain consistency, growth media for plates were solidified using 2% bacteriological agar from US Biological (catalog no.: A0930). Anaerobic cultures were maintained in O_2_-depleted culture jars (GasPak EZ Container Systems; BD Diagnostics). The Bi-SC plates for visualizing sulfite reductase activity were made as previously described (69, 70). Briefly, the plates contained (w/v) 0.67% yeast nitrogen base, 0.2% SC(-Ura/-Met) dropout mix, 1% β-alanine, 0.1% bismuth ammonium citrate, 0.3% sodium sulfite, and 2% glucose. The sulfide (S^2-^) produced by sulfite reductase reacts with bismuth (Bi^3+^) to produce the brown precipitate bismuth(III) sulfide (Bi_2_S_3_).

The *gsh1Δ* strains were constructed by deleting the *GSH1* gene using the *GSH1* replacement plasmid pGSH1KO-HIS3 (see later). GSH deplete (*gsh1Δ*) strains were selected on SC(-His) + 25 μM GSH following transformation with pGSH1KO-HIS3. Successful deletion of *GSH1* was confirmed by colony PCR and/or Western blot using anti-Gsh1 antibodies (69). Following plasmid transformation, *gsh1Δ* strains were routinely maintained anaerobically on SC + 0.05–0.1 μM GSH glucose selection plates to provide the minimum GSH necessary for growth while avoiding carryover of stored GSH into subsequent spot tests and liquid growth assays.

### Plasmid construction and transformation

The *GSH1* deletion plasmid (pGSH1KO-HIS3) was prepared by cloning upstream and downstream regions of the *GSH1* gene using the primers listed in Table S2. The upstream PCR product was digested with BamHI and EcoRI and the downstream with SalI and EcoRI. Both digested PCR products were ligated in a trimolecular reaction into the BamHI and SalI sites of the yeast integrating plasmid pRS403 (*HIS3* selection). After linearization with EcoRI, the integrating deletion plasmid was inserted into the yeast genome *via* homologous recombination. The p416TEF-*HGT1* plasmid described previously was used for *HGT1* overexpression (9). The p315TEF-*HGT1* plasmid was created by inserting a DNA fragment amplified from p416TEF-*HGT1* with the *TEF1* promoter, *HGT1* coding sequence, and *CYC1* terminator into pRS315 digested with EagI and SacI. The cytosol-pHluorin expression plasmid pJH700 was created by moving the *ACT1* promoter, pHluorin coding sequence, and *CYC1* terminator from pYES2-*P*_*ACT1*_-pHluorin into pRS415 digested with SpeI and SacII. All plasmid sequences were confirmed by Sanger sequencing (Genewiz). Yeast transformations were performed by standard lithium acetate protocols (71). Plasmids used in this study are listed in Table S3.

### Growth assays in liquid and solid media

Liquid growth assays were performed in sterile 96-well plates using a Synergy H1 plate-reader and Gen5 Software 2.09. Cells were started at absorbance of 0.05 at 600 nm, and wells were sealed with a Breathe-Easy sealing membrane (Sigma–Aldrich). Cells were grown at 30 °C with continuous orbital shaking (559 rpm), and the absorbance at 600 nm was measured every 30 min. For solid media spot tests, cells were harvested from SC(-Ura) + 0.05–1 μM GSH plates after 1 to 3 days of anaerobic growth at 30 °C and serially diluted in MQH_2_O to 1.0, 0.1, 0.01, and 0.001 absorbance at 600 nm. The resuspended cells were spotted on SC or SMM selection plates and incubated at 30 °C for 2 to 3 days either aerobically or anaerobically.

### Subcellular fractionation

Yeast cells were grown aerobically to midlog phase in SC selection medium with 2% glucose. Mitochondrial and post-mitochondrial supernatant (PMS) fractions were obtained as previously described by converting cells to spheroplasts followed by gentle lysis using a loose-fitting Dounce homogenizer and differential centrifugation (72). Incubation with DTT during the spheroplasting step was omitted to avoid reduction of endogenous disulfides. Protein content was measured using the Bradford assay with bovine serum albumin as a calibration standard.

### GSH–GSSG assay

Yeast whole-cell lysates for measurement of total GSH (GSH + GSSG) were made according to published methods (40). Measurement of total GSH in acidified extracts was performed with the 5,5-dithiobis(2-nitrobenzoic acid)-GSSG reductase cycling assay using a microplate reader following a published protocol (73).

### Western blots

Yeast extracts subjected to gel electrophoresis were analyzed by Western blotting using anti-Gsh1 polyclonal antibodies (69). Anti-phosphoglycerate kinase (Pgk1) antibodies (Invitrogen) were used as a loading control. Western blots were visualized using an Odyssey Infrared Imaging System (LI-COR).

### Redox Western blots, intracellular pH measurements, and redox potential calculations

Redox Western blot analysis of cytosol-rxYFP was performed as previously described *via* nonreducing SDS-PAGE (74). Reduced and oxidized forms of rxYFP were analyzed by quantitative immunoblot using an Odyssey Infrared Imaging System (LI-COR). Intracellular pH measurements with cytosol-pHluorin were performed according to a published protocol (15). Cells were grown in low fluorescence SC selection media (Sunrise Pharmaceuticals). The cytosol-pHluorin sensor was first calibrated *in situ* in each strain by resuspending cells in citrate-phosphate buffer (McIlvaine’s buffer) ranging in pH from 4.8 to 8.4. Fluorescence emission of pHluorin was measured at 512 nm using a Biotek Synergy H1 plate reader with excitation at 390 nm and 470 nm. Background fluorescence for a WT strain not expressing pHluorin was subtracted from the measurements. The ratio of emission intensity (R_390_/R_470_) was calculated and plotted against the corresponding buffer pH. The data were fit to the following sigmoidal curve function using GraphPad Prism: $R=R_{\min}+\frac{\left(R_{\max}-R_{\min}\right)}{1+10^{\left(pK_{a}-pH\right)}}$ where *R* is the ratio of fluorescence emission intensities for excitation at 390 nm and 470 nm (R_390_/R_470_) at a given pH, *R*_*min*_ is the ratio (R_390_/R_470_) measured for cells in the most acidic (pH 4.8) buffer, and *R*_*max*_ is the ratio (R_390_/R_470_) measured for cells in the most alkaline (pH 8.4) buffer. The constant p*K*_a_ was determined from the inflection point of the curve. Individual pH values were calculated from experimental *R* readings using *R*_*max*_, *R*_*min*_, and p*K*_a_ from the calibration curves and the following equation: $\text{pH}=pK_{a}+\log\left[\frac{\left(R-R_{\min}\right)}{\left(R_{\max}-R\right)}\right]$.

The cytosolic GSH:GSSG redox potential at 30 °C was calculated using the Nernst equation adjusted for pH as follows:

$$E=E{{}^{\circ}}^{\prime }+\left[-60.1\;\text{mV}*\left(pH-7.0\right)\right]-\left[\frac{60.1\;\text{mV}}{2}\log\frac{\left(100-\%\;\text{ox}\right)}{\%\;\text{ox}}\right]$$

where *Eº′* is the redox potential of rxYFP at pH 7.0 (−265 mV) (75), pH is the cytosolic pH measured with cytosol-pHluorin, and % ox is the % oxidized rxYFP measured from the rxYFP redox Western blot.

### Subcellular Fe analysis and Fe–S cluster enzyme assays

All Fe measurements and Fe–S enzyme activity assays were performed on strains grown in SC(-Ura) glucose media. Fe levels in cytosolic (PMS) and mitochondrial extracts were measured using a PerkinElmer PinAAcle 900T graphite furnace atomic absorption spectrometer according to the manufacturer’s instructions. Cytosolic and mitochondrial fractions were prepared as described previously, and Fe content was standardized per milligram protein. Succinate dehydrogenase, aconitase, and isopropylmalate isomerase enzyme activities were assayed as previously described (76, 77). For the qualitative analysis of sulfite reductase activity, Bi-SC plates were made as described previously, and yeast strains were serially diluted and spotted at 1.0, 0.1, 0.01, and 0.001 absorbance at 600 nm.

### *FIT2pr*-GFP Aft1 reporter assay

Aft1 reporter yeast strains containing the *FIT2pr*-GFP transgene and p315TEF empty vector or p315TEF-*HGT1* overexpression plasmid were grown in low fluorescent SC(-Leu) glucose media overnight to midlog phase and harvested for experiments as previously described (17). During this overnight growth, the *gsh1Δ* + vector strain underwent five to six doublings before growth arrest because of critical depletion of GSH stores. GFP fluorescence was measured using a Biotek Synergy H1 plate reader with 485 nm excitation and 528 nm emission wavelengths.

### RNA isolation and quantitative RT–PCR analysis

BY4741 WT and *gsh1Δ* strains transformed with control vector (p416TEF) or *HGT1* overexpression plasmid (p416TEF-HGT1) were grown overnight to midlog phase in SC(-Ura) with no added GSH. As noted previously, the *gsh1Δ* + vector strain underwent five to six doublings before reaching growth arrest. Total RNA was isolated from yeast strains using the PureLink RNA Mini kit (Invitrogen) following the manufacturer’s protocol using the lysis buffer supplied by the kit. Yeast RNAs were reverse transcribed into complementary DNAs with iScript Reverse (Bio-Rad) following the manufacturer’s recommendations. Using the primers shown in Table S2, quantitative RT–PCR was carried out on an IQ ICycler (Bio-Rad) with the SsoAdvanced SYBR Green Supermix kit (Bio-Rad). The fold change in *FET3* and *FIT3* expression, normalized to *CMD1* (calmodulin) and relative to the WT + vector control, was calculated by the 2^-ΔΔC^^T^ method (78).

### Statistical analysis

Data were analyzed using GraphPad Prism, version 10.3.1 for Mac (GraphPad Software). Comparisons between two groups were performed using unpaired, parametric Student’s *t* test with Welch correction. *p* Values on all graphs are as follows: ns, not significant, ∗*p* < 0.05, ∗∗*p* < 0.01, ∗∗∗*p* < 0.001, ∗∗∗∗*p* < 0.0001.

## Data availability

All data are included in the article.

## Supporting information

This article contains supporting information. (9, 10, 14, 15, 16, 17, 19, 75, 79, 80)

## Conflict of interest

The authors declare there are no conflicts of interest with the contents of this article.
