# Supplementary material for: Cysteine import via the high-affinity GSH transporter Hgt1 rescues GSH auxotrophy in yeast
Source: J Biol Chem. 2024 Dec 21;301(2):108131. doi: 10.1016/j.jbc.2024.108131 (PMC11786745; doi:10.1016/j.jbc.2024.108131)
Supplement: Supporting information [file mmc1.pdf]

# Supporting Information

# Cysteine Import via the High Affinity Glutathione Transporter Hgt1 Rescues Glutathione Auxotrophy in Yeast

Crystal C. McGee‡, Tirthankar Bandyopadhyay‡, Cailin N. McCracken, Evan Talib, Courtney E. Patterson, Caryn E. Outten*

Department of Chemistry and Biochemistry, University of South Carolina, Columbia, SC, USA

‡These authors contributed equally to this work.

*Corresponding author: outten@sc.edu

**Table S1.** Yeast strains used in this study.

**Table S2.** Primers used in this study.

**Table S3.** Plasmids used in this study.

**Figure S1**. Growth of WT and *gsh1*Δ strains with or without *HGT1* overexpression with increasing GSSG.

**Figure S2.** Redox and pH measurements with rxYFP and cytosol-pHluorin in WT and *gsh1*Δ strains ± *HGT1* overexpression.

**Figure S3.** Homocysteine and N-acetyl cysteine rescue growth of *gsh1*Δ + *HGT1* strains in the absence of GSH.

**Figure S4.** Cysteine rescue of *gsh1*Δ + *HGT1* strains is observed in cells grown without GSH for >72 hrs and in other yeast backgrounds.

**Figure S5.** Hgt1 transporter activity is required for robust rescue of *gsh1*Δ + *HGT1* strains by cysteine.

**Table S1.** Yeast strains used in this study.

| **Yeast Strain** | **Genotype** | **Source** |
|---|---|---|
| BY4741 WT | *MATa, his3Δ1, leu2Δ0, met15Δ0, ura3Δ0* | Open Biosystems |
| BY4741 *gsh1*Δ | *BY4741 gsh1*Δ*::HIS3* | This study |
| BY4741 Aft1 reporter | BY4741 chrI(199456-199457)::$P_{FIT2}$-yeGFP-$Term_{ADH1}$:KanMX | (1) |
| BY4741 *gsh1*Δ Aft1 reporter | BY4741 Aft1 reporter *gsh1Δ*::*HIS3* | This study |
| BY4741 *met10* | BY4741 *met10*Δ*::kanMX4* | Open Biosystems |
| BY4742 WT | *MATα, his3Δ1, leu2Δ0, lys2Δ0, ura3Δ0* | Open Biosystems |
| BY4742 *gsh1*Δ | *BY4742 gsh1*Δ*::HIS3* | This study |
| DY150 WT | *MATa ade2-1 his3-11 leu2-3,112 trp1-1 ura3-52 can1-100(oc)* | Gift from Jerry Kaplan |
| DY150 *gsh1*Δ | *DY150 gsh1*Δ*::HIS3* | This study |

**Table S2.** Primers used in this study. Restriction enzyme sites used for cloning are underlined.

| **Primer Name** | **Primer Sequence** | **Restriction Enzyme** |
|---|---|---|
| GSH1KO Primer A | GATTATATTGAATTCTTGTGCTGGAG | EcoRI |
| GSH1KO Primer B | GATTCCACGGATCCTTAATG | BamHI |
| GSH1KO Primer C | GATTTGCTGTCGACGTGTGATAG | SalI |
| GSH1KO Primer D | CTCAGAGATCTTTGAATTCTTGTTC | EcoRI |
| FIT3 RT-PCR forward | TCCGCTTTGGTTCTATCTGC | |
| FIT3 RT-PCR reverse | AGTGCTGCTGGCGTAAGAGT | |
| FET3 RT-PCR forward | TCACAGTTTCGATCCGGACAACCA | |
| FET3 RT-PCR reverse | TCCACGAGAACAAGACCCAAACCT | |
| CMD1 RT-PCR forward | CGCCCAGTGAAGCAGAAGTAAATG | |
| CMD1 RT-PCR reverse | ATCTCGCCTGATCCATCACTAACC | |

**Table S3.** Plasmids used in this study.

| **Primer Name** | **Description** | **Source** |
|---|---|---|
| pGSH1KO(HIS3) | *GSH1* knockout cassette in pRS403 (YIp, *HIS3*) | This study |
| p416TEF-HGT1 | *HGT1* gene driven by *TEF1* promoter in pRS416 (*CEN URA3)* | (2) |
| p315TEF-HGT1 | *HGT1* gene driven by *TEF1* promoter in pRS315 (*CEN LEU2)* | This study |
| p416TEF-YCT1 | *YCT1* gene driven by *TEF1* promoter in pRS416 (*CEN URA3)* | (3) |
| pHOJ150 | Cytosol-rxYFP expression driven by *PGK1* in pRS304 (*YIp LEU2*) | (4) |
| pYES2-$P_{ACT1}$ -pHluorin | Cytosol-pHluorin expression driven by *ACT1* in pYES2 (2µ *URA3*) | (5) |
| pJH700 | Cytosol-pHluorin expression driven by *ACT1* in pRS415 (*CEN LEU2*) | This study |

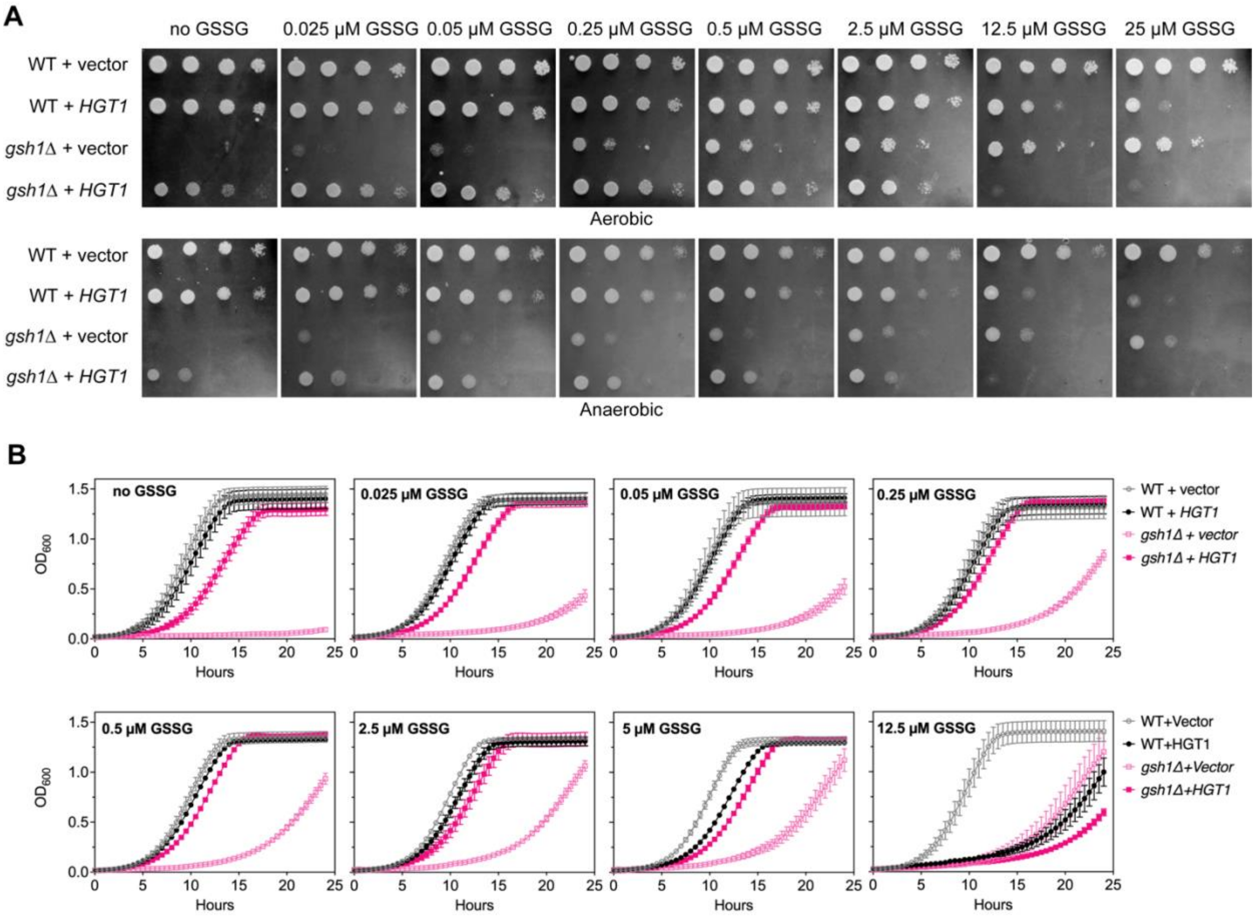


**Figure S1. Growth of WT and *gsh1*Δ strains with or without *HGT1* overexpression with increasing GSSG.** (A) Ten-fold serial dilutions of WT (BY4741) and isogenic *gsh1*Δ strains transformed with empty vector (p416TEF) or *HGT1* overexpression plasmid (p416TEF-HGT1) were plated on SC(-Ura) glucose media with varying GSSG concentrations and grown aerobically (top) or anaerobically (bottom). Each GSSG is converted to 2 GSH by glutathione reductase in cells. (B) The indicated yeast strains were grown in SC(-Ura) glucose media with the indicated GSSG concentrations and $OD_{600}$ measurements were taken every 30 minutes for 24 hrs. The growth curves are reported as means of 3 biological replicates ± SD.

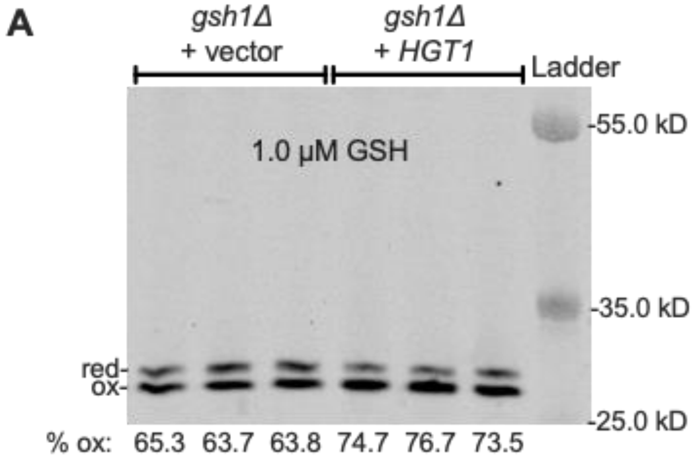


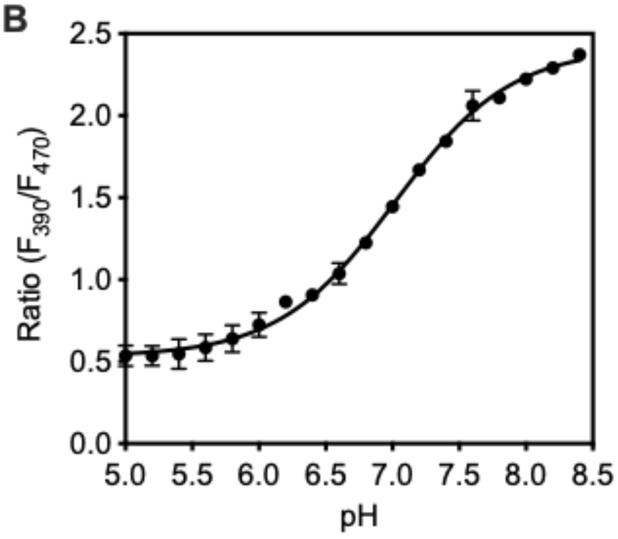


| | pH | SD |
|---|---|---|
| **WT + vector** | 7.30 | 0.03 |
| **WT + *HGT1*** | 7.40 | 0.03 |
| ***gsh1Δ*+ vector** | 7.33 | 0.03 |
| ***gsh1Δ*+ *HGT1*** | 7.35 | 0.05 |

**Figure S2. Redox and pH measurements with rxYFP and cytosol-pHluorin in WT and *gsh1*Δ strains ± *HGT1* overexpression.** (A) Representative redox western blot of *gsh1*Δ + vector and *gsh1*Δ + *HGT1* strains transformed with cytosol-rxYFP expression plasmid (pHOJ150) to measure the cytosolic GSH:GSSG redox state. Three biological replicates for each strain grown in SC(-Leu /-Ura) glucose media with 1 μM GSH were separated by non-reducing SDS-PAGE and immunoblotted with anti-GFP antibodies. Positions of reduced and disulfide-oxidized rxYFP are shown on the left. Numbers below each lane indicate % oxidized rxYFP determined from band quantification. The prestained protein ladder is loaded in the last lane. **(**B**)** Calibration curve for cytosol-pHluorin expressed in WT + *HGT1* cells resuspended in buffers of varying pH values as described in the Experimental Procedures. The curve was fit using the Henderson-Hasselbalch equation yielding p$K_a$ = 7.02 ± 0.05 (6). Similar calibrations curves and p$K_a$ values were obtained for pHluorin expressed in WT + vector, *gsh1*Δ + vector, and *gsh1*Δ + *HGT1* strains. Table at right shows intracellular pH values measured using cytosol-pHluorin for the indicated strains following overnight growth in SC(-Leu/-Ura) media. Average pH values and standard deviation (SD) are given for 6 biological replicates. The % oxidized rxYFP and cytosolic pH values were used to calculate the GSH:GSSG redox potentials shown in Fig. 3.

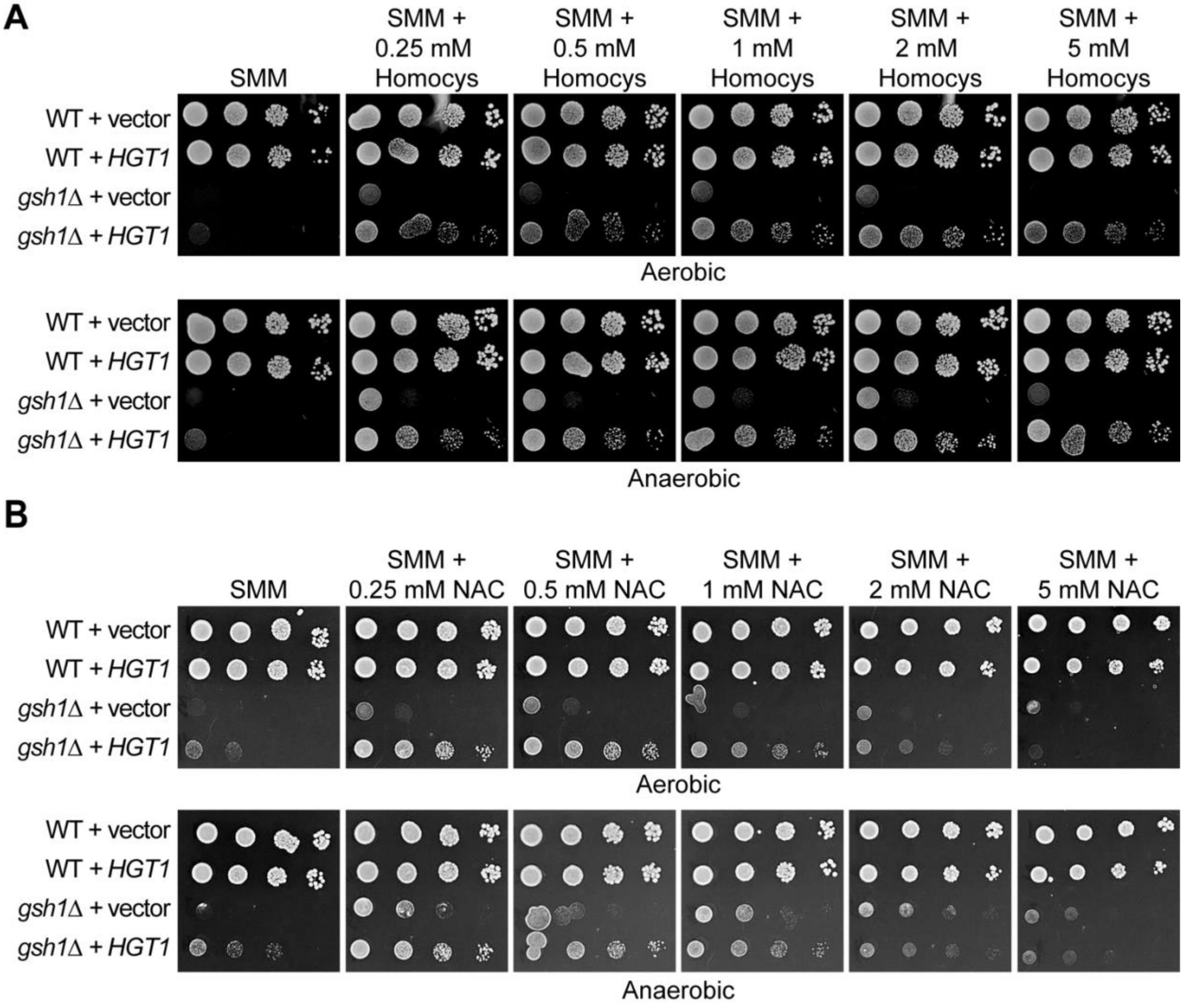


**Figure S3. Homocysteine and N-acetyl cysteine rescue growth of *gsh1*Δ + *HGT1* strains in the absence of GSH.** (A) Ten-fold serial dilutions of WT (BY4741) and isogenic *gsh1*Δ strains transformed with empty vector (p416TEF) or *HGT1* overexpression plasmid (p416TEF-HGT1) were plated on SMM glucose media with the indicated concentrations of homocysteine (A) or N-acetylcysteine (NAC) (B) added to the media.

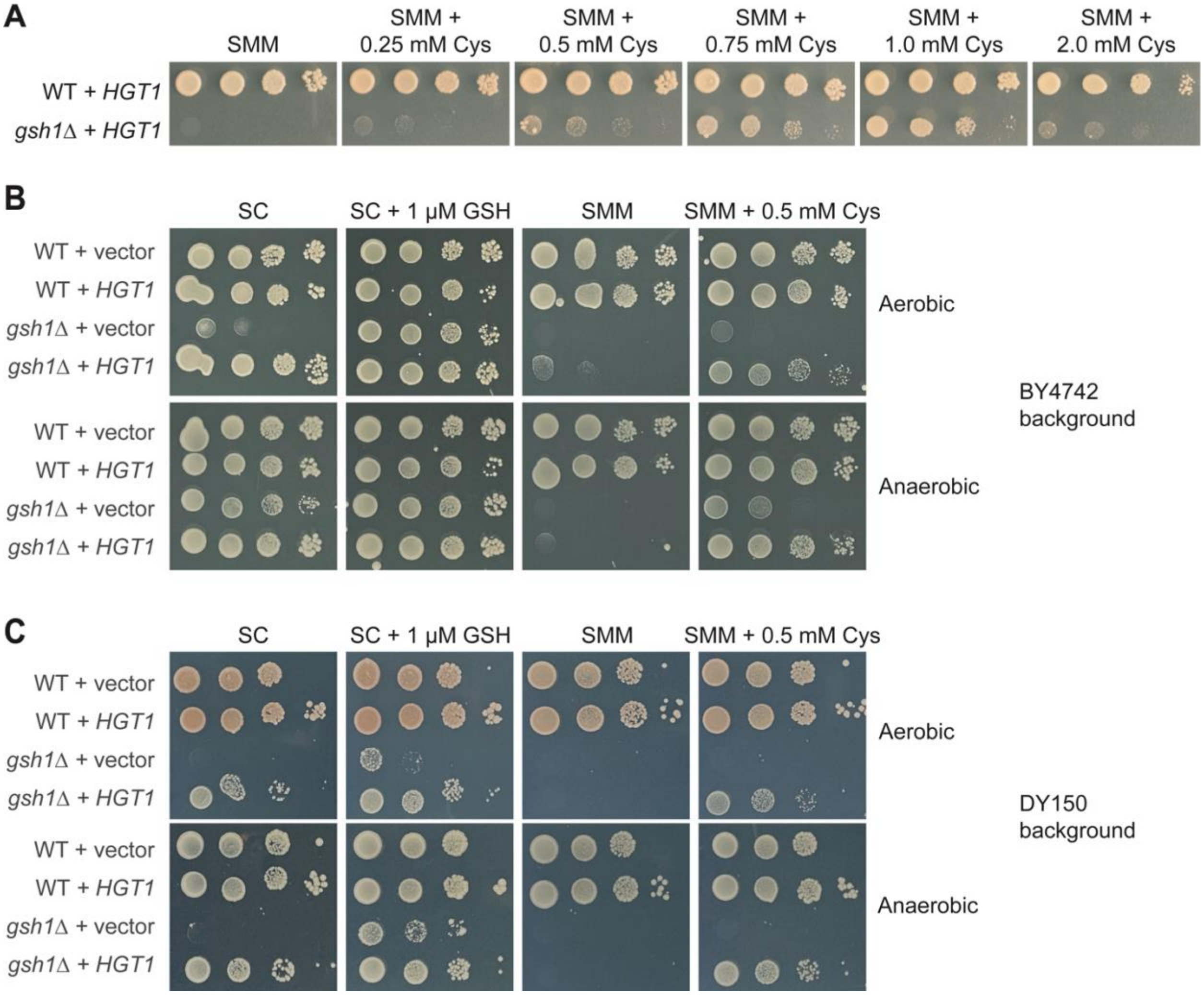


**Figure S4. Cysteine rescue of *gsh1*Δ + *HGT1* strains is observed in cells grown without GSH for >72 hrs and in other yeast backgrounds.** (A) WT (BY4741) and isogenic *gsh1*Δ strains transformed with p416TEF-HGT1 were pre-grown in liquid media for 72 hours in SC-Ura glucose media, with dilution every 24 hours to maintain exponential growth. The cells were then serially diluted to 1, 0.1, 0.01, and 0.001 $OD_{600}$ and spotted on SMM(-Ura) media with increasing cysteine concentrations and grown anaerobically for 2-3 days at 30 °C. (B,C) Growth of WT and *gsh1*Δ BY4742 (B) and DY150 (C) yeast strains with or without *HGT1* overexpression. For (B), ten-fold serial dilutions of WT and isogenic *gsh1*Δ strains transformed with empty vector (p416TEF) or *HGT1* overexpression plasmid (p416TEF-HGT1) were plated on SC(-Ura) glucose media with or without 1 µM GSH or SMM(-Ura) glucose media with or without 0.5 mM cysteine and grown aerobically (top) or anaerobically (bottom). Similar results were obtained using *LEU2* selection plasmids and -Leu plates (data not shown). For (C), *LEU2* plasmids (p315TEF and p315TEF-HGT1) were used with -Leu plates. We note that unlike BY4741 and BY4742, the WT DY150 colonies are reddish-colored because this strain is an *ade2* mutant that accumulates a red pigment in adenine-limiting medium in the presence of oxygen and GSH (7, 8). This color is not apparent in DY150 WT strains grown anaerobically and DY150 *gsh1*Δ strains that lack sufficient GSH.

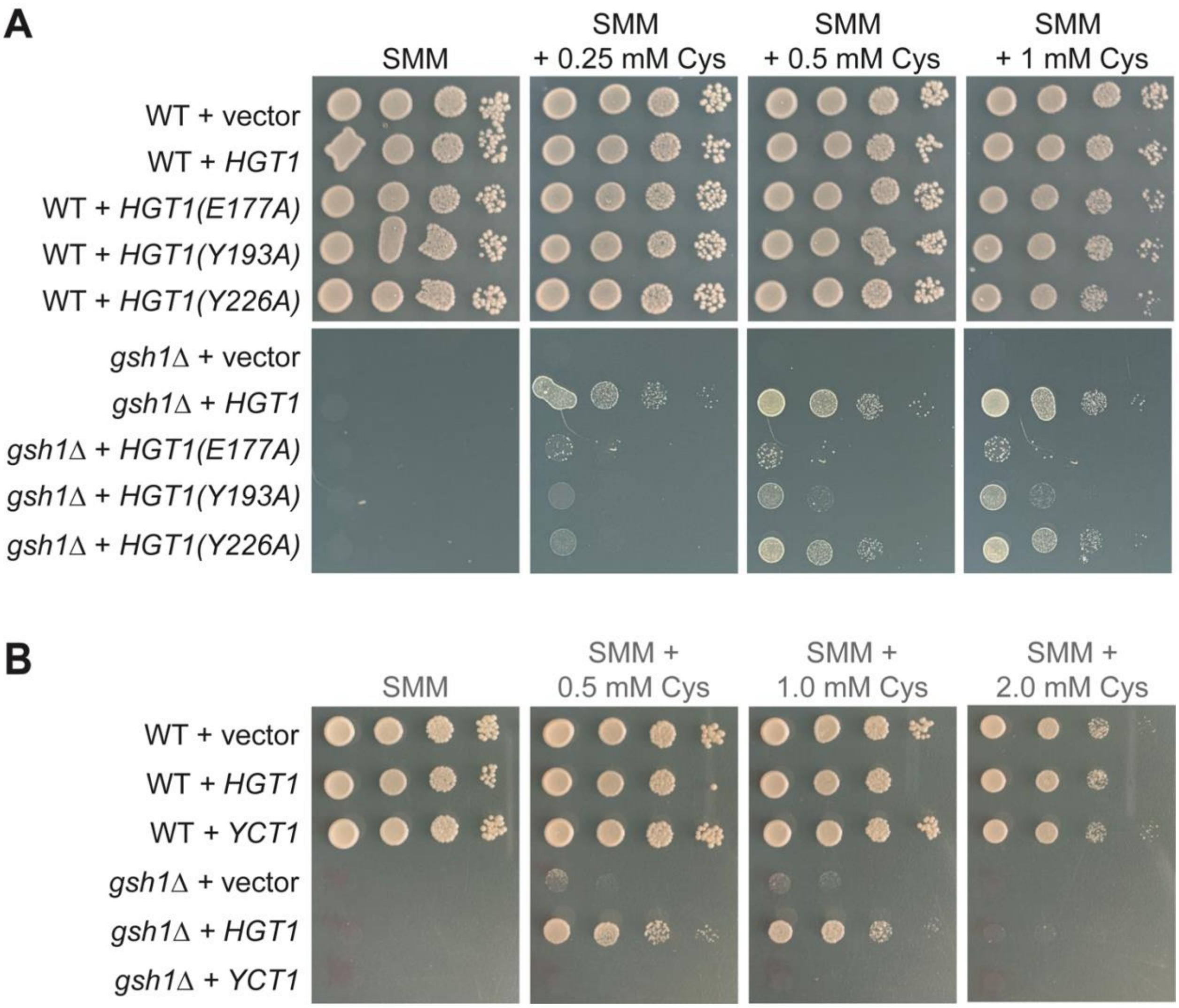


**Figure S5. Hgt1 transporter activity is required for robust rescue of *gsh1*Δ + *HGT1* strains by cysteine.** Ten-fold serial dilutions of WT (BY4741) and isogenic *gsh1*Δ strains transformed with empty vector (p416TEF), *HGT1* overexpression plasmids (p416TEF-HGT1(WT), p416TEF-HGT1(E177A), p416TEF-HGT1(Y193A), or p416TEF-HGT1(Y226A)) or *YCT1* overexpression plasmid (p416TEF-YCT1) were plated on SMM(-Ura) glucose media with increasing cysteine as indicated and grown aerobically for 2-3 days at 30 °C. (A) Comparison of *HGT1* mutants in WT and *gsh1*Δ strains. The *HGT1* mutants tested were previously shown to have normal expression levels and localize properly to the plasma membrane (9, 10). (B) Comparison of *HGT1* vs. *YCT1* overexpression in WT and *gsh1*Δ strains.

## References


1. Hughes, C. E., Coody, T. K., Jeong, M. Y., Berg, J. A., Winge, D. R., and Hughes, A. L. (2020) Cysteine toxicity drives age-related mitochondrial decline by altering iron homeostasis. *Cell* **180**, 296-310 e218

2. Bourbouloux, A., Shahi, P., Chakladar, A., Delrot, S., and Bachhawat, A. K. (2000) Hgt1p, a high affinity glutathione transporter from the yeast *Saccharomyces cerevisiae*. *J. Biol. Chem.* **275**, 13259-13265

3. Kaur, J., and Bachhawat, A. K. (2007) Yct1p, a novel, high-affinity, cysteine-specific transporter from the yeast *Saccharomyces cerevisiae*. *Genetics* **176**, 877-890

4. Østergaard, H., Tachibana, C., and Winther, J. R. (2004) Monitoring disulfide bond formation in the eukaryotic cytosol. *J. Cell Biol.* **166**, 337-345

5. Orij, R., Postmus, J., Ter Beek, A., Brul, S., and Smits, G. J. (2009) In vivo measurement of cytosolic and mitochondrial pH using a pH-sensitive GFP derivative in *Saccharomyces cerevisiae* reveals a relation between intracellular pH and growth. *Microbiology* **155**, 268-278

6. Miesenbock, G., De Angelis, D. A., and Rothman, J. E. (1998) Visualizing secretion and synaptic transmission with pH-sensitive green fluorescent proteins. *Nature* **394**, 192-195

7. Sharma, K. G., Kaur, R., and Bachhawat, A. K. (2003) The glutathione-mediated detoxification pathway in yeast: an analysis using the red pigment that accumulates in certain adenine biosynthetic mutants of yeasts reveals the involvement of novel genes. *Arch. Microbiol.* **180**, 108-117

8. Smirnov, M. N., Smirnov, V. N., Budowsky, E. I., Inge-Vechtomov, S. G., and Serebrjakov, N. G. (1967) Red pigment of adenine-deficient yeast *Saccharomyces cerevisiae*. *Biochem. Biophys. Res. Commun.* **27**, 299-304

9. Zulkifli, M., and Bachhawat, A. K. (2017) Identification of residues critical for proton-coupled glutathione translocation in the yeast glutathione transporter, Hgt1p. *Biochem. J.* **474**, 1807-1821

10. Zulkifli, M., Yadav, S., Thakur, A., Singla, S., Sharma, M., and Bachhawat, A. K. (2016) Substrate specificity and mapping of residues critical for transport in the high-affinity glutathione transporter Hgt1p. *Biochem. J.* **473**, 2369-2382
